# Sustainable Hydrogels in Water Treatment—A Short Review

**DOI:** 10.3390/gels11100812

**Published:** 2025-10-10

**Authors:** Anita Ioana Visan, Irina Negut

**Affiliations:** National Institute for Lasers, Plasma and Radiation Physics, 077125 Măgurele, Romania; anita.visan@inflpr.ro

**Keywords:** hydrogel, pollution removal, green synthesis, heavy metals

## Abstract

The growing worldwide water shortage, intensified by pollution from industrial and human activities, highlights the urgent need for advanced, eco-friendly water treatment solutions. Hydrogels, which are three-dimensional polymer networks with exceptional water absorption capabilities, are gaining attention as effective materials for purification, thanks to their remarkable absorption, selectivity, and reusability. This review offers a concise introduction to hydrogels, focusing on their sustainable aspects such as biodegradability, minimal toxicity, and sourcing from renewable materials. We emphasize their benefits compared to traditional treatment approaches and outline the key goals of this review: categorizing and analyzing the synthesis, modification, characteristics, and varied uses of sustainable hydrogels in eliminating inorganic and organic contaminants. Additionally, we explore their potential for regeneration, current limitations, and future prospects in alignment with environmental sustainability.

## 1. Introduction

Hydrogels are versatile polymeric materials known for their ability to absorb and retain large amounts of water within their three-dimensional (3D) network without dissolving [1]. Their tunable mechanical properties, porosity, and swelling capacity make them ideal for water treatment, enabling efficient filtration and adsorption of contaminants like heavy metals, dyes, and organic pollutants [2].

A key advantage of hydrogels is their environmental sustainability. Many are biocompatible, biodegradable, and non-toxic, offering a greener alternative to conventional materials [3]. Traditional water treatment methods often rely on non-biodegradable materials, contributing to pollution [4], whereas cellulose-based hydrogels that are derived from abundant natural polymers provide a renewable, low-cost, and high-performance solution [5].

Hydrogels excel in multiple water treatment applications. Their porous structure allows effective adsorption of pollutants through physical entrapment and chemical interactions [6]. They also enhance membrane-based purification and desalination by improving selectivity, permeability, and antifouling properties [7]. Functional modifications (e.g., adding carboxyl or amino groups) further optimize their adsorption capacity and pollutant specificity [8].

**Figure 1:**
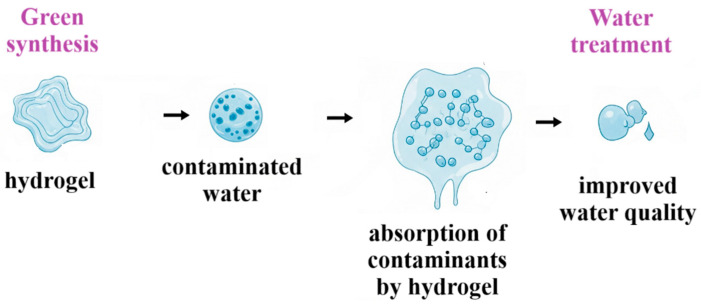
Schematic representation of the green synthesis of natural polymer for the absorption of contaminants, leading to effective pollution removal.

The growing pollution from industrialization and agriculture increases the need for advanced solutions [9]. Conventional methods face limitations like high costs and secondary waste [10], while hydrogels offer high efficiency, even at low pollutant concentrations [11]. Sustainable hydrogel development prioritizes renewable biomass sources, green synthesis, and biodegradability, aligning with circular economy principles [12]. Figure 1 illustrates a comprehensive approach to water treatment using green synthesis and hydrogels, highlighting their environmental benefits.

While significant literature exists on the use of hydrogels for water treatment, a comprehensive perspective that integrates sustainability across the entire hydrogel lifecycle remains underexplored. Previous studies have primarily focused on isolated aspects, such as the efficiency of pollutant removal [13], the synthesis from specific natural polymers [14], or the regeneration of a spent hydrogel [15]. However, there is a critical research gap in synthesizing these findings to address the full lifecycle, from the use of renewable, eco-friendly synthesis routes to ensuring end-of-life biodegradability and reusability. The existing body of work also falls short in addressing current limitations, such as the scalability of synthesis methods and performance in complex, multi-component wastewater streams. To advance this field, there is a clear need to explore future prospects, including AI-driven design for optimized hydrogel properties and the pilot-scale implementation of these materials to validate their real-world feasibility and cost-effectiveness [16].

## 2. Design and Synthesis of Sustainable Hydrogels

The design and synthesis of sustainable hydrogels for water treatment focuses on utilizing renewable, biodegradable, and environmentally friendly materials while optimizing performance for water purification. Key strategies include selecting natural polymers such as cellulose, chitosan [17], alginate [18], lignin [19], and gelatin [20], which offer biocompatibility, biodegradability, and abundant functional groups for pollutant adsorption and water retention [21,22].

**Figure 2:**
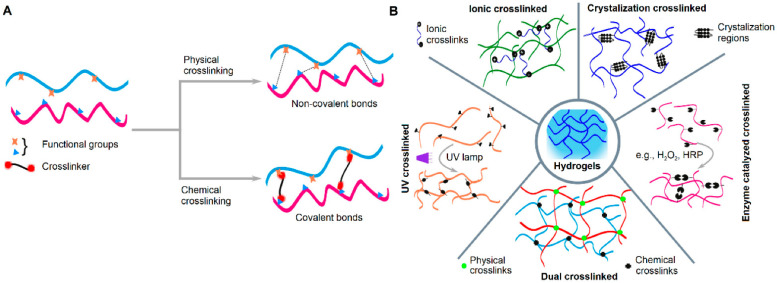
Representation showing (**A**) the effect of physical and chemical crosslinking on the type of bonds formed and (**B**) several examples of different crosslinking techniques [23].

Crosslinking is the essential process that forms the three-dimensional network in hydrogels by connecting individual polymer chains. This process can be achieved through two primary mechanisms: physical crosslinking and chemical crosslinking (Figure 2).

Physical crosslinking involves the formation of temporary, reversible bonds between polymer chains. These bonds are non-covalent, relying on weaker interactions such as hydrogen bonding, ionic interactions, and hydrophobic forces. Because these bonds are reversible and can be broken by changes in temperature, pH, or ionic strength, the resulting hydrogels can be dissolved or “un-gelled.” Physical methods avoid toxic reagents and are therefore considered more biocompatible, though they typically result in lower mechanical strength and stability [24].

Chemical crosslinking, in contrast, creates a permanent and irreversible network by forming strong covalent bonds between polymer chains. This process requires a crosslinking agent that reacts with the polymer chains, often initiated by heat, UV light, or catalysts. The resulting structure is stable and resistant to dissolution but may raise concerns about residual crosslinking agents and potential toxicity [25].

The two methods produce hydrogels with distinct properties:

(i) Stability and reusability: Physically crosslinked hydrogels are reversible and less stable, making them suitable for applications like on-demand drug release. Chemically crosslinked hydrogels are irreversible and highly stable, making them ideal for long-term biomedical or industrial use [16,21,24,25].
(ii) Mechanical strenght: Chemical crosslinking generally produces hydrogels with superior mechanical strength and rigidity due to covalent bonds, while physically crosslinked hydrogels tend to be softer and more elastic [16,21,24,25].
(iii) Toxicity: Physical crosslinking is typically more biocompatible because it avoids the use of chemical reagents that could introduce toxic impurities, whereas chemical crosslinking requires careful purification [16,21,24,25].
(iv) Synthesis complexity: Physical crosslinking methods are simpler, requiring only environmental triggers, while chemical crosslinking is more complex and requires specific reagents and controlled reaction parameters [16,21,24,25].

The incorporation of functional additives, such as nanoparticles or hydrophilic groups, further enhances adsorption, selectivity, and reusability, while the network structure is engineered for optimal porosity and mass transport [16,25,26]. Recent advances also emphasize the use of plant-derived nanomaterials and inspiration from natural hierarchical structures to improve water interactions and hydrogel performance [22]. Sustainable hydrogels are designed to be easily degradable or recyclable after use, supporting circular economy principles and reducing secondary pollution [17,27].

Overall, the synthesis of these hydrogels integrates material selection, crosslinking technology, and functionalization to create efficient, eco-friendly platforms for water treatment applications [16,21,22,26,28]. The design and synthesis of sustainable hydrogels are critical for their effective and environmentally responsible application in water treatment. This section explores the choice of raw materials, green synthesis routes, and functionalization strategies that enhance their performance and align with sustainability principles.

### 2.1. Raw Materials

**Figure 3:**
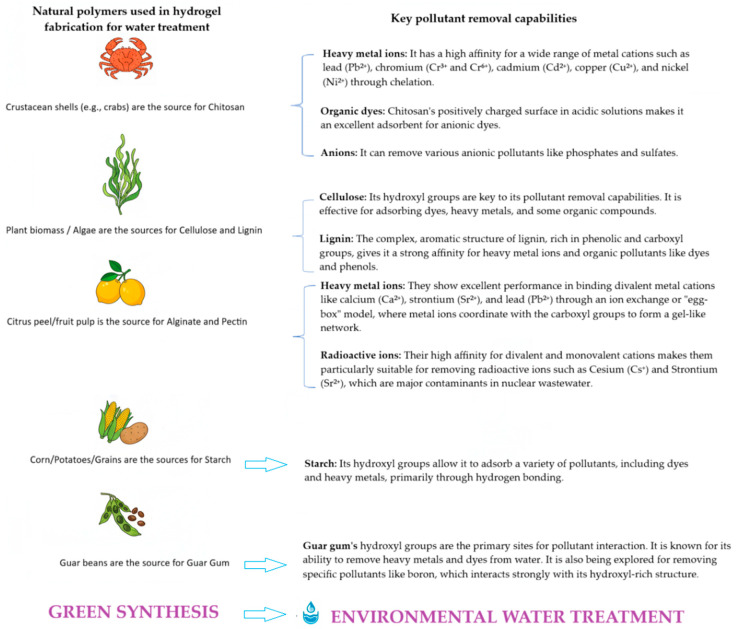
Natural polymers used in hydrogel fabrication for water treatment, highlighting their sources (e.g., crustacean shells for chitosan, plants for cellulose; lignin; alginate, pectin, starch, guar gum) and key pollutant removal capabilities (e.g., heavy metals, anionic dyes, oxyanions).

The choice of raw materials is a critical determinant of the sustainability, performance, and environmental impact of hydrogels used in water treatment [29]. A paradigm shift towards natural, renewable, and biodegradable polymers has gained interest, determined by the need to reduce reliance on petroleum-based synthetic materials and to minimize ecological harm [30]. Natural polymers, including chitosan [31], cellulose [29], alginate [29], starch [32], guar gum [33], and pectin [34], serve as sustainable raw materials for hydrogels, offering distinct advantages such as biodegradability, renewable sourcing, and intrinsic functional groups for pollutant removal [35] (Figure 3).

This next section evaluates key materials, their advantages, and challenges in sustainable hydrogel design.

#### 2.1.1. Natural Polymers: Renewable and Biocompatible Alternatives

Natural polymers derived from renewable resources have emerged as highly attractive materials for developing sustainable hydrogels for water treatment applications [36].

Among these, chitosan stands out as a particularly valuable biopolymer obtained from chitin, which is abundantly present in crustacean shells and fungal biomass [37]. The presence of amine (–NH_2_) and hydroxyl (–OH) functional groups in chitosan enables effective removal of various pollutants through multiple mechanisms including chelation, electrostatic interactions, and ion exchange [38]. This cationic polysaccharide demonstrates remarkable efficiency in adsorbing anionic dyes such as Congo Red and Methyl Orange, as well as heavy metal ions including Cu^2+^, Cr^6+^, and Pb^2+^ [39]. Beyond its adsorption capabilities, chitosan possesses inherent antimicrobial properties that make it valuable for microbial disinfection applications [40]. However, the mechanical weakness of pure chitosan hydrogels often necessitates cross-linking with agents like glutaraldehyde or blending with nanomaterials such as graphene oxide to enhance their stability and performance in water treatment systems [41].

Cellulose, as the most abundant natural polymer on Earth, offers exceptional potential for hydrogel development due to its high porosity, mechanical strength, and numerous modifiable hydroxyl groups [42]. Derived from plant sources like wood and cotton or produced by bacteria, cellulose can be processed into nanocellulose forms including cellulose nanocrystals (CNC) and cellulose nanofibrils (CNF) that provide dramatically increased surface area for pollutant adsorption [43]. These nanocellulose materials have shown particular effectiveness in removing heavy metal ions such as Cd^2+^, Ni^2+^, and Pb^2+^ from contaminated water [44]. When functionalized and combined with advanced materials like graphene oxide or carbon nanotubes, cellulose-based hydrogels exhibit enhanced compressive strength and excellent regeneration efficiency [45]. Bacterial cellulose, with its unique ultra-fine fibrous network structure, represents another promising variant that demonstrates superior performance in water purification applications [45].

Alginate, a polysaccharide extracted from brown seaweed, has gained significant attention for its ability to selectively bind divalent metal ions through its carboxylate (–COO^−^) functional groups [46]. The ionotropic gelation properties of alginate, typically achieved using Ca^2+^ ions, allow for relatively simple and scalable hydrogel production under mild conditions [47]. Various forms of alginate hydrogels, including fibrous structures, aerogels, and composite membranes, have proven effective for heavy metal removal from aqueous solutions [48]. However, the mechanical fragility of alginate hydrogels in aqueous environments remains a limitation that often requires reinforcement strategies such as incorporation of nanomaterials or blending with synthetic polymers to improve their durability in practical water treatment applications [49].

Among plant-derived polymers, starch, guar gum, and pectin have shown considerable promise for developing sustainable hydrogels. Starch, obtained from abundant sources like corn, potatoes, and cassava, forms hydrogels with tunable hydrophilicity that are particularly effective for dye removal due to their porous structure and reactive hydroxyl groups [50]. Guar gum, a galactomannan derived from guar beans, produces hydrogels with exceptional water absorption capacity that can effectively absorb pollutants like Cr^6+^ and various dyes while also providing viscosity-enhancing properties that contribute to hydrogel stability [51]. Pectin, rich in carboxyl groups and obtained from citrus peels and apple pomace, forms hydrogels through cross-linking with Ca^2+^ or Fe^3+^ ions and has demonstrated excellent performance in removing Pb^2+^ and Cu^2+^ ions from water, with the added benefits of being completely biodegradable and non-toxic [52].

Lignin, as the second most abundant biopolymer after cellulose and a major byproduct of the pulp and paper industry, represents a highly promising yet underutilized resource for sustainable hydrogel development [53]. This complex aromatic polymer contains numerous functional groups including hydroxyl, carboxyl, and methoxyl groups that enable effective pollutant removal through various interaction mechanisms [54]. Lignin-based hydrogels offer several advantages including enhanced mechanical strength due to lignin’s rigid structure [55], inherent antioxidant and antimicrobial properties that can inhibit biofilm formation, and high adsorption capacity for both dyes and heavy metal ions [56]. However, challenges remain in working with lignin due to its heterogeneous nature and dark coloration, which may cause secondary pollution if not properly processed, requiring careful optimization of extraction and modification techniques [57].

The use of these natural polymers for hydrogel development offers several significant advantages from a sustainability perspective. Their inherent biodegradability helps minimize persistent environmental waste and supports circular economy principles [36], while their renewable nature reduces dependence on finite fossil resources and lowers the overall carbon footprint compared to petroleum-based polymers [58]. Many of these biopolymers also possess intrinsic functional groups that can directly interact with pollutants, potentially reducing the need for complex or harsh modification procedures [59]. However, several challenges must be addressed to fully realize their potential, including the mechanical weakness of many natural polymer hydrogels that often requires cross-linking or nanocomposite reinforcement [59], batch-to-batch variability due to the natural sourcing of these materials [60], and scalability limitations associated with some extraction and processing methods [61]. Future advancements in green chemistry approaches and AI-driven material design are expected to further optimize these natural polymer hydrogels for large-scale water treatment applications [62].

#### 2.1.2. Synthetic Polymers: Balancing Performance and Sustainability

While synthetic polymers have traditionally raised concerns regarding their persistence, toxicity, and environmental impact, certain polymers, such as polyvinyl alcohol (PVA) [63], polyacrylic acid (PAA) [64], and polyacrylamide (PAM) [65], remain highly relevant due to their superior water retention, mechanical strength, and precisely tunable chemistry [66]. In order to mitigate their environmental footprint, several strategies have been developed, including hybrid networks, green synthesis methods, and precise functionalization.

Combining synthetic polymers like PVA or PAA with natural polymers (e.g., chitosan-PAA [67] or cellulose-PVA [68] significantly enhances biodegradability while preserving the advantageous properties of synthetic polymers. For example, PAA-based hydrogels can achieve exceptional swelling capacities, with water uptake exceeding 1000%, while maintaining durability for repeated adsorption–desorption cycles [69]. These hybrid systems leverage the strengths of both synthetic and natural polymers, improving performance while reducing environmental impact [70].

To minimize hazardous chemical use and energy consumption, green synthesis methods such as solvent-free reactions [70], radiation-induced polymerization (e.g., UV, electron beams, gamma radiation) [71], and enzyme-assisted crosslinking have been developed. These approaches reduce waste generation and improve the sustainability of synthetic polymer production [16].

A key advantage of synthetic polymers is their ability to be precisely functionalized for specific applications. For instance, PAA can be tailored to effectively remove cationic dyes or heavy metal ions such as Ni^2+^, Cu^2+^, and Co^2+^ from contaminated water [72]. This level of customization enhances pollutant removal efficiency, making synthetic polymers highly effective in water treatment applications.

Synthetic polymers offer several key benefits in environmental applications. Their high durability and mechanical strength allow them to withstand rigorous operational conditions and repeated use, extending their functional lifespan [73]. Additionally, their precise and versatile functionality enables the introduction of specific functional groups, optimizing pollutant adsorption efficiency [74]. Furthermore, synthetic polymers are highly scalable and reproducible, making them suitable for large-scale manufacturing with consistent quality [75].

Despite their advantages, synthetic polymers present notable challenges. Non-degradability is a major concern, as many synthetic polymers persist in the environment, contributing to microplastic pollution unless designed with hydrolyzable bonds for controlled degradation [76]. Another issue is the potential toxicity of monomers and precursors, such as acrylamide in PAA synthesis, which requires stringent handling and purification to ensure safety [77]. Additionally, the production of synthetic polymers often relies on petroleum-derived feedstocks, leading to a higher carbon footprint compared to natural alternatives [78].

While synthetic polymers present environmental challenges, advancements in hybrid networks, green synthesis, and precise functionalization help mitigate their impact. Their superior mechanical properties, tunability, and scalability make them indispensable in water treatment applications, provided that sustainability considerations are addressed in their design and production [78].

To further enhance sustainability and align with circular economy principles, ongoing research explores the valorization of waste-derived materials as precursors for hydrogel synthesis. One such material is lignin, a major byproduct of the paper and pulp industries. Rich in phenolic and hydroxyl groups, lignin shows great promise as a precursor for hydrogels used in heavy metal binding and dye removal [79].

Additionally, protein-based materials, such as soy protein and gelatin, derived from agricultural or food processing waste, can be utilized to create hydrogels. These often offer pH-responsive networks, allowing for controlled pollutant capture and release, and have shown promise in removing heavy metals like Cd^2+^ and Pb^2+^ [80]. Gelatin, for example, has been used to form hydrogels with enhanced mechanical properties [81]. Algal and biomass polysaccharides, upcycled from various agro-industrial wastes or renewable algal biomass, serve as low-cost and abundant feedstocks for sustainable hydrogel production [82]. Furthermore, fly ash, an industrial waste product from thermal power plants, has been explored as a cost-effective, unconventional cross-linking agent in the synthesis of hydrogels for heavy metal removal [82].

The future of sustainable hydrogels lies in optimizing hybrid systems that merge the eco-friendliness and inherent functionalities of natural polymers with the enhanced performance and durability of selected synthetics. This also involves vigorously advancing circular-economy feedstocks derived from waste streams. This integrated approach will lead to water treatment solutions that are both highly effective and environmentally responsible [83].

Table 1 provides a comprehensive overview of various hydrogel raw materials, detailing their origins, key functional groups, target pollutants, and a balanced perspective on their sustainability.

### 2.2. Green Synthesis Routes

Traditional hydrogel synthesis methods often rely on a variety of techniques that, while effective, can pose significant environmental and health risks. These conventional approaches typically involve the use of toxic chemical crosslinkers such as glutaraldehyde and N,N′-methylenebis(acrylamide) [14], which can leach from the final product and raise concerns about biocompatibility and potential toxicity. Furthermore, the synthesis process frequently requires organic solvents that are volatile, flammable, and harmful to the environment [15]. Many of these methods are also energy-intensive, demanding high temperatures or prolonged reaction times, which contributes to a larger carbon footprint [92].This reliance on hazardous chemicals and energy-demanding processes provides a stark contrast to the emerging green synthesis routes, highlighting why a shift towards more sustainable and eco-friendly alternatives is not only beneficial but crucial for the advancement of hydrogel technology.

The synthesis of environmentally sustainable hydrogels requires methods that minimize hazardous chemicals, energy consumption, and waste generation while maintaining high performance. Traditional hydrogel fabrication often relies on toxic crosslinkers, organic solvents, and energy-intensive processes, which contradict sustainability goals [93,94]. This section highlights four key green synthesis strategies that align with circular economy principles. Each green synthesis method offers distinct advantages and trade-offs concerning reaction time, energy use, scalability, and applicability to different material types [95]. Table 2 provides a concise comparison of the green synthesis methods discussed, highlighting their key characteristics and optimal applications.

Next, we will discuss the mentioned green synthesis routes, being is crucial for developing sustainable hydrogels, offering a path to minimize environmental impact while maximizing performance in diverse applications.

#### 2.2.1. Solvent-Free Synthesis

Solvent-free synthesis eliminates the use of organic solvents like DMF, THF, or ethanol. This significantly reduces toxicity, simplifies purification processes, and minimizes environmental pollution from solvent waste [100].

This approach includes several key methods. Melt polymerization involves crosslinking thermoplastic polymers, such as PVA or starch, under heat without needing solvents, allowing for direct processing [101]. Solid-state reactions entail the mechanical mixing of dry precursors, like chitosan and citric acid, followed by thermal curing, which reduces liquid waste streams [102].

The advantages of solvent-free synthesis are clear: it creates no solvent waste and avoids complex solvent recovery, leading to reduced operational costs and environmental burden. These methods are also potentially scalable for industrial production due to their simpler processes, and they enhance safety in manufacturing environments. For example, starch-PVA hydrogels crosslinked with maleic acid in melt form have been effectively used for dye adsorption without generating any solvent residues [103].

#### 2.2.2. Microwave-Assisted Synthesis

Microwave-assisted synthesis works by using microwave irradiation to speed up chemical reactions through dielectric heating. In this process, polar molecules, such as water or monomers, absorb microwave energy and directly convert it into heat [104]. This quick, focused heating can significantly cut down reaction times and energy consumption by over 50% when compared to traditional heating methods [105].

This technique has several important applications. It is great for graft copolymerization, as it quickly grafts monomers onto polymer backbones. For example, you can synthesize cellulose-graft-poly(acrylamide) hydrogels in minutes, a process that would take hours with conventional methods [106]. It also helps with nanocomposite hydrogels by promoting an even dispersion of nanoparticles (like clay, TiO_2_, or graphene oxide) within the polymer matrix. This happens because of the fast and selective heating, which boosts the composite’s synergistic properties [107].

The advantages of microwave-assisted synthesis are numerous from which we mention faster kinetics and higher yields, meaning drastically reduced reaction times and improved productivity and efficiency [108]. It is also energy-efficient due to the fact that its targeted molecular activation, leading to lower overall energy use. Plus, it can enhance product quality, resulting in more uniform polymer networks and better control over hydrogel properties [109]. However, there is a challenge: for very large-scale batches, the limited penetration depth of microwaves can become a limiting factor, potentially leading to uneven heating [104].

#### 2.2.3. Bio-Derived Crosslinkers and Physical Crosslinking

This strategy centers on replacing synthetic, often toxic, crosslinking agents like glutaraldehyde, N,N’-methylenebis(acrylamide) (MBA), or epichlorohydrin. Instead, it uses renewable, non-toxic alternatives or leverages physical interactions to form stable hydrogel networks. This approach significantly boosts the biocompatibility and biodegradability of the final product [110].

There are two main types of crosslinking under this principle. Natural chemical crosslinkers include genipin, a natural compound derived from Gardenia fruits, known for crosslinking proteins and polysaccharides (like chitosan) with significantly lower cytotoxicity (e.g., 10 times lower) than glutaraldehyde [111]. Citric acid and tannins, which are rich in carboxyl groups, can enable esterification or hydrogen bonding with polysaccharides to form stable networks. Additionally, enzymatic crosslinking uses enzymes such as laccase or transglutaminase to catalyze crosslinking reactions under mild conditions [112].

The other type is physical crosslinking, which relies on non-covalent interactions, often making these hydrogels thermoreversible or pH-responsive. Examples include freeze–thaw cycling, where repeated freezing and thawing of polymer solutions (e.g., PVA, chitosan) creates crystallites or hydrogen bonds that act as physical crosslinks, leading to robust hydrogels [113]. Ionic gelation uses multivalent ions to crosslink charged polymers; for instance, alginate easily gels with Ca^2+^ ions, and chitosan can gel with polyanions like tripolyphosphate (TPP) [114,115]. Finally, hydrophobic associations involve the self-assembly of hydrophobic domains within block copolymers or grafted polymers to form physical junctions [116].

The advantages of this strategy are substantial: it leads to enhanced biocompatibility and biodegradability by avoiding toxic residues from synthetic crosslinkers, making hydrogels safer for environmental and potential biomedical applications. It also reduces environmental impact by using renewable resources and minimizing hazardous waste generation. Furthermore, it offers tunable reversibility, as physically crosslinked hydrogels can often be dissolved and reformed, which helps with regeneration and recycling. For example, chitosan-genipin hydrogels have shown high efficiency (e.g., >90% adsorption) for Cr(VI) removal, all while being fully biodegradable and less toxic [115]. Similarly, PVA-chitosan hydrogels created via a freeze–thawing method have demonstrated exceptional mechanical properties and anti-swelling characteristics [116].

#### 2.2.4. Radiation-Induced Polymerization

In radiation-induced polymerization, high-energy radiation, such as UV light, gamma rays, or electron beams, directly starts polymerization and crosslinking reactions without needing chemical initiators or catalysts [117]. This process works by creating free radicals along the polymer chains, which then react to form a strong, crosslinked network.

There are a few key techniques within this method. UV curing is a fast and efficient option, often used for acrylate-functionalized polymers like poly(ethylene glycol) diacrylate (PEGDA), which can crosslink in mere seconds under UV light. Gamma and electron beam irradiation involve high-energy radiation that can break polymer chains to generate radicals. This allows for both direct crosslinking and the grafting of monomers onto existing polymer backbones, such as cellulose with acrylic acid [118]. A bonus of these methods is that they also offer sterilization benefits during synthesis.

Radiation-induced polymerization offers several notable advantages, the most significant being the absence of residual chemical initiators in the final product. This eliminates concerns related to the presence of unreacted initiators such as ammonium persulfate (APS) or tetramethyl ethylenediamine (TEMED), as well as unpolymerized monomers, thereby enhancing the purity and biocompatibility of the synthesized material [119]. This method also provides precise control over the crosslinking density and hydrogel properties by simply adjusting the radiation dose [120]. Finally, it is a single-step synthesis that can lead to the rapid and efficient creation of solid hydrogels under mild operating conditions. For instance, gamma-irradiated carboxymethyl cellulose hydrogels have been successfully used to remove Pb^2+^, achieving high adsorption capacities of up to 500 mg/g [121].

Furthermore, process intensification, through the development of continuous flow systems for hydrogel synthesis, can enhance efficiency and scalability, further reducing the environmental footprint. Lastly, waste valorization remains a key area. Expanding the range of waste-derived precursors and developing efficient conversion pathways will be essential for achieving fully circular material economies in hydrogel production [26].

### 2.3. Functionalization Strategies

**Figure 4:**
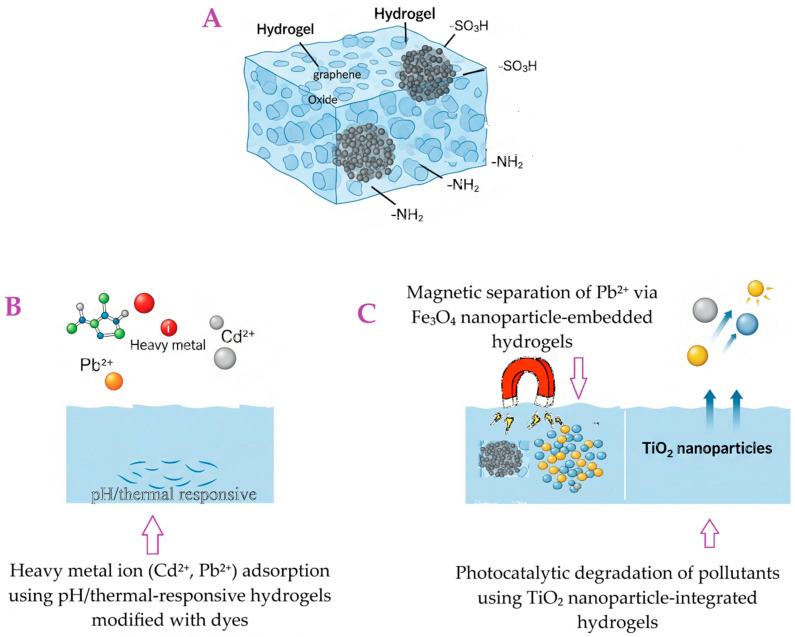
Schematic illustration of functionalized hydrogels for environmental applications: (**A**) A general hydrogel structure, a porous, three-dimensional network. Embedded within this hydrogel matrix are particles of graphene oxide. The hydrogel incorporates graphene oxide to enhance its mechanical stability and adsorption capacity. The hydrogel itself is shown to be functionalized with various chemical groups, indicating its potential for chemical interactions. (**B**) A magnified, molecular-level view of a section of the hydrogel from Panel A. Key functional groups (–SOH, –COOH, –NH_2_) grafted onto the hydrogel network, enabling selective pollutant binding. (**C**) Heavy metal ion (Cd^2+^, Pb^2+^) adsorption using pH/thermal-responsive hydrogels modified with dyes.

Functionalization is a crucial aspect of tailoring hydrogel properties for specific pollutant removal applications. It allows for enhanced selectivity, improved adsorption capacity, and better reusability of the hydrogels. Figure 4 illustrates the diverse functionalization strategies employed in hydrogel design, showcasing how their inherent properties are tailored for efficient pollutant removal and environmental responsiveness.

One key aspect of functionalization is grafting for enhanced selectivity. This involves introducing specific functional groups onto the polymer backbone, which significantly improves the hydrogel’s affinity for target pollutants. For instance, carboxylic groups (–COOH) and sulfonic acid groups (–SO_3_H) are effective at enhancing the affinity for heavy metals through chelation and ion exchange [122]. Similarly, amino groups (–NH_2_) are highly effective for capturing anionic dyes or metal ions [123]. Various grafting methods, including chemical, radiation, or enzymatic techniques, are used to increase the number of available adsorption sites and enhance surface polarity.

Another important approach is the creation of nanocomposite hydrogels. This involves integrating nanomaterials such as clays (e.g., bentonite, montmorillonite), graphene oxide (GO), carbon nanotubes (CNTs), or metal/metal oxide nanoparticles (e.g., TiO_2_, Fe_3_O_4_, Ag) into the hydrogel matrix [124]. This integration imparts synergistic properties, leading to enhanced mechanical strength, increased adsorption capacity, and sometimes even catalytic activity, as seen with the photocatalytic degradation of dyes using TiO_2_ [125]. The inclusion of magnetic nanoparticles, like Fe_3_O_4_, is particularly beneficial as it allows for easy separation of the adsorbent from treated water using an external magnetic field, thus addressing a major challenge in adsorbent recovery [126].

The synthesized and functionalized hydrogels are characterized by several critical properties that directly influence their performance in water treatment. Swelling capacity refers to their ability to absorb large volumes of water, which is essential for efficient pollutant capture; this capacity is influenced by factors such as cross-linking density, pH, and temperature [127]. Mechanical stability is crucial for ensuring the hydrogels can withstand various operational conditions and maintain their structural integrity, especially for repeated use [128]. Some hydrogels exhibit pH/thermal responsiveness, meaning they are designed to react to changes in pH or temperature, allowing for controlled release of adsorbed pollutants during regeneration cycles [129]. Biodegradability is the capacity of the hydrogel to break down into non-toxic byproducts after its lifespan, minimizing long-term environmental impact [130]. Lastly, regeneration potential highlights the ability of the hydrogels to be reused for multiple cycles of adsorption and desorption, which ultimately reduces waste and operational costs [131].

### 2.4. Characterization Techniques

A comprehensive understanding of hydrogels’ properties and performance requires the integration of advanced analytical and imaging techniques. These approaches provide complementary information on the chemical structure, morphology, and thermal stability of hydrogels, thereby guiding their optimization for environmental, biomedical, and industrial applications.

To provide a clearer overview, the following table (Table 3) organizes the wide range of techniques into four major categories: physicochemical, analytical (drug detection and quantification), drug diffusion, and biological evaluation. This categorization highlights how each method contributes unique insights, from understanding chemical structure and stability to assessing release kinetics and in vivo performance.

No single technique suffices to characterize hydrogel drug delivery systems. Instead, a multimodal strategy, combining physicochemical analyses, advanced substance quantification methods, diffusion studies, is essential. Such integrated characterization ensures reproducibility, enhances understanding of release mechanisms, and accelerates clinical translation

## 3. Applications in Water Treatment

Sustainable hydrogels offer truly versatile solutions for tackling a wide array of water pollutants, demonstrating remarkably high efficacy in various treatment scenarios [13].

Table 4 provides specific examples demonstrating the practical application and performance of various hydrogel systems in removing different types of pollutants.

### 3.1. Removal of Inorganic Pollutants

Hydrogels are proving to be particularly effective in removing toxic inorganic contaminants, primarily through highly efficient adsorption mechanisms. This is crucial for the protection of both environmental and human health [139].

#### 3.1.1. Heavy Metals

Among the most hazardous and prevalent water pollutants are heavy metals such as Pb^2+^, Cd^2+^, As^3+^, Cr^6+^, Cu^2+^, Ni^2+^, and Hg^2+^. Hydrogels can be specifically engineered, or “functionalized,” with groups like -COOH, -NH_2_, or -SH [140]. These groups actively facilitate adsorption through powerful chelation, ion exchange, and electrostatic interactions [122]. For instance, hydrogels based on chitosan show an exceptional affinity for a broad spectrum of heavy metals, largely due to the protonation of their amine groups in acidic conditions, which enhances binding [123]. Furthermore, the integration of nanocomposite materials like graphene oxide or various metal oxides (e.g., iron oxide, Fe_3_O_4_) into the hydrogel structure can dramatically boost removal efficiency and, importantly, enable easy separation of the loaded adsorbent from the treated water [126,138,141]. Studies consistently show that these advanced hydrogels can achieve impressive adsorption capacities, often exceeding 500 mg/g, significantly outperforming many conventional, less sustainable adsorbents currently in use [142].

A sodium alginate/coffee waste composite hydrogel achieved 98.4% removal of Pb(II) from aqueous solutions, with high reusability over multiple cycles. The hydrogel’s carboxyl and hydroxyl groups facilitated strong adsorption via ion exchange and chelation mechanisms [135].

A bifunctional hydrogel synthesized from modified starch demonstrated maximum adsorption capacities of 350 mg/g for Co^2+^ ions, outperforming many conventional adsorbents. The adsorption followed pseudo-second-order kinetics, indicating chemisorption as the dominant mechanism [136].

**Figure 5:**
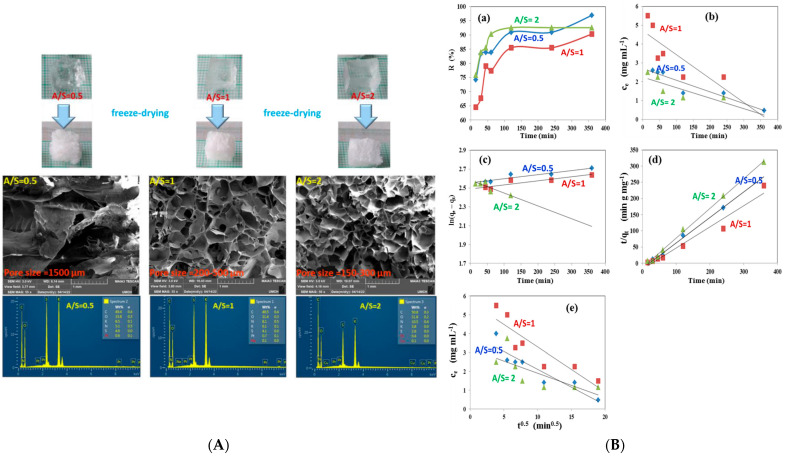
(**A**) he prepared HPMC-g-poly(AM-co-SPA) hydrogels before and after freeze-drying; SEM images of their porous morphology; and the EDX elemental analysis of these hydrogels; (**B**) (**a**) Effect of contact time, (**b**) fit (zero-order reaction), (**c**) pseudo-first-order rate, (**d**) pseudo-second-order rate, and (**e**) intra-particle diffusion [137].

Tohami et al. developed hydrogels based on hydroxypropyl methyl cellulose (HPMC) grafted with acrylamide (AM) and 3-sulfopropyl acrylate (SPA), synthesized through radical polymerization initiated by the oxidation-activated HPMC backbone (Figure 5). The grafted copolymer chains were crosslinked into a 3D network using a small amount of divinyl comonomer. HPMC was selected for its affordability, hydrophilicity, and natural origin, while AM and SPA were incorporated to selectively bind coordinating and cationic inorganic pollutants, respectively. The resulting hydrogels exhibited strong elasticity and high mechanical strength, with stress at break reaching several hundred percent. In Cr(VI) adsorption tests, the hydrogels demonstrated high removal efficiency (90–96%) in a single step (Figure 4). Particularly, hydrogels with AM/SPA ratios of 0.5 and 1 showed potential for regeneration through pH adjustment, making them suitable for repeated Cr(VI) [137].

**Figure 6:**
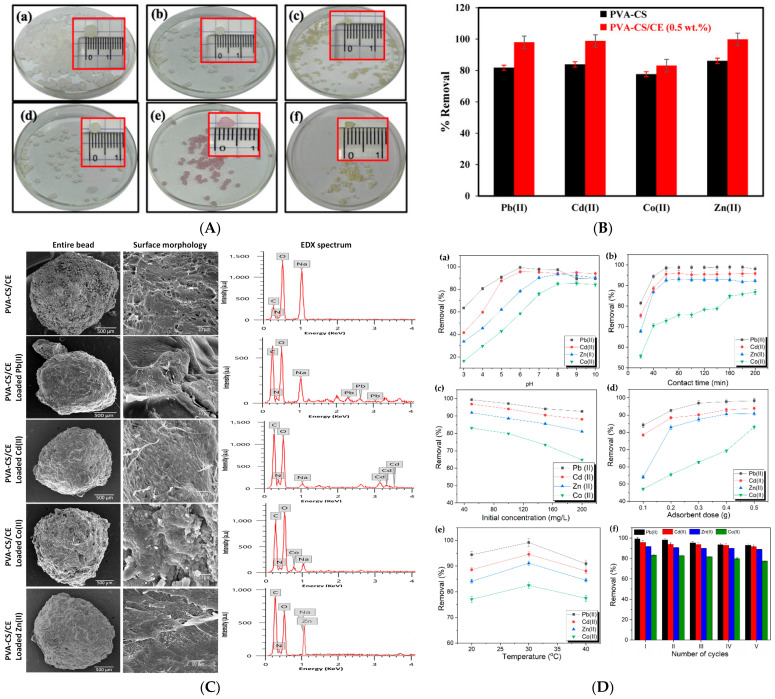
(**A**) PVA-CS/CE composite hydrogel beads (**a**) before drying, (**b**) after 48 h of air drying, as well as dried beads after adsorption of (**c**) Pb(II), (**d**) Cd(II), (**e**) Co(II), or (**f**) Zn(II). Insets show the size of beads; (**B**) Effect of CE content (0, and 0.5 wt.%) on percent removal of Pb(II), Cd(II), Co(II), and Zn(II) at hydrogel beads (C_0_ [heavy metals] = 50 mg/L, volume = 50 mL, pH (Pb and Cd) = 6, pH (Co and Zn) = 8, bead dose = 0.5 g, time = 200 min, temperature = 30 °C); (**C**) SEM images of the entire surface of PVA-CS/CE hydrogel beads before and after adsorption of heavy metal ions as well as EDX spectra of PVA-CS/CE hydrogel beads; (**D**) Effects of different factors on the removal efficiency of Pb(II), Cd(II), Zn(II), and Co(II) onto PVA-CS/CE composite hydrogel beads: (**a**) pH (C_o_[Metals] = 50 mg/L, V = 50 mL, bead dose = 0.5 g, pH = 3–10, t = 200 min, T = 30 °C), (**b**) Contact time (C_o_[Metals] = 50 mg/L, V = 50 mL, pH = 6 (Pb and Cd) and pH = 8 (Zn and Co), bead dose = 0.5 g, T = 30 °C), (**c**) Initial concentration of metals (V = 50 mL, pH = 6 (Pb and Cd) and pH = 8 (Zn and Co), bead dose = 0.5 g, t = 200 min, T = 30 °C), (**d**) Adsorbent dose (C_o_[Metals] = 50 mg/L, V = 50 mL, pH = 6 (Pb and Cd) and pH = 8 (Zn and Co), t = 200 min, T = 30 °C), (**e**) Temperature (C_o_[Metals] = 50 mg/L, V = 50 mL, pH = 6 (Pb and Cd) and pH = 8 (Zn and Co), bead dose = 0.5 g, t = 300 min), and (**f**) Reusability (C_o_[Metals] = 50 mg/L, V = 50 mL, pH = 6 (Pb and Cd) and pH = 8 (Zn and Co), bead dose = 0.5 g, t = 200 min, T = 30 °C) [143].

In another study, researchers utilized poly(vinyl alcohol) (PVA), chitosan (CS), and cellulose (CE) to fabricate a composite hydrogel adsorbent (PVA-CS/CE) through a simple physical crosslinking process. This method was designed for the efficient removal of heavy metal ions—Pb(II), Cd(II), Zn(II), and Co(II)—from aqueous solutions. The influence of various adsorption parameters, including pH, contact time, adsorbent dosage, initial metal ion concentration, and temperature, was systematically investigated. The adsorption behavior followed the pseudo-second-order kinetic model and aligned well with the Langmuir isotherm model, indicating monolayer chemisorption. The PVA-CS/CE hydrogel exhibited high removal efficiencies for Pb(II), Cd(II), Zn(II), and Co(II), achieving 99%, 95%, 92%, and 84% removal, respectively, within 60 min (Figure 6). The study also highlighted that the hydrated ionic radius of metal ions significantly influenced adsorption selectivity. Notably, after five adsorption–desorption cycles, the material retained over 80% of its removal efficiency, suggesting strong potential for reuse in industrial wastewater treatment applications [143].

#### 3.1.2. Nutrient Recovery

Excess nitrates (NO_3_^−^) and phosphates (PO_4_^3−^) originating from agricultural runoff and urban wastewater are notorious for causing eutrophication in water bodies, leading to algal blooms and oxygen depletion. Hydrogels, especially those derived from chitosan and other biopolymers, can effectively capture and remove these harmful nutrients. For example, specific hydrogels like zirconium-crosslinked carboxymethyl cellulose/chitosan have shown enhanced phosphate removal capabilities, primarily through ligand exchange and electrostatic attraction. Nitrate removal can also occur effectively via hydrogen bonding interactions, particularly at lower pH levels. This ability to recover nutrients not only cleans water but also opens avenues for resource recovery.

Starch-based hydrogels grafted with functional groups such as 2-acrylamido-2-methylpropane-1-sulphonic acid (AMPS) exhibited phosphate adsorption capacities up to 650 mg/g. The removal mechanism involved ligand exchange and electrostatic attraction, particularly effective at low pH [141]. Polysaccharide-based composite hydrogels, including those with chitosan and cellulose, have been used for nitrate and phosphate removal from agricultural runoff, helping to mitigate eutrophication. These hydrogels can be regenerated and reused, supporting sustainable nutrient management [144].

**Figure 7:**
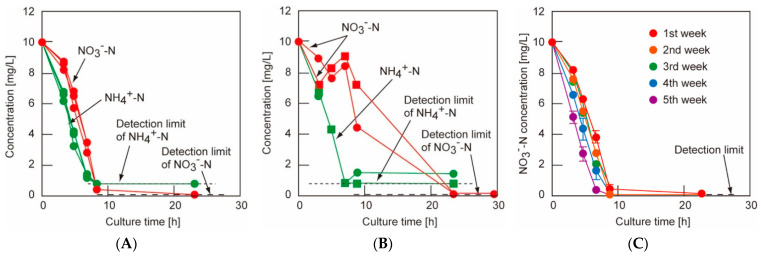
(**A**) Comparison of nitrogen removal between denitrification of nitrate nitrogen and nitrification of ammonia nitrogen by alginate beads: red circle, nitrate-nitrogen concentration, green circle, ammonia-nitrogen concentration. The detection limit of nitrate nitrogen by the ion chromatography method is 0.2 mg/L. The alginate beads were prepared by adding 1 mL of *R. gra* suspension to 10 mL of 1.5% concentration sodium alginate solution. The detection limit of nitrate nitrogen by the ion chromatography method is 0.2 mg/L, and that of ammonia nitrogen by the indophenol blue method is 0.8 mg/L; (**B**) Simultaneous nitrogen removal of nitrate nitrogen and ammonia nitrogen by alginate beads: red symbols, nitrate-nitrogen concentration, green symbols, ammonia-nitrogen concentration, Circle and square symbols indicate first run and second run, respectively. The detection limit of nitrate nitrogen by the ion chromatography method is 0.2 mg/L, and that of ammonia nitrogen by the indophenol blue method is 0.8 mg/L; (**C**) Repeated denitrification properties of alginate hydrogel beads with *R. gra* [145].

The heterotrophic nitrification–aerobic denitrification (HN-AD) process offers a promising strategy for nitrate-nitrogen removal from water. In this study, Rhodotorula graminis NBRC0190, a naturally occurring red yeast with strong nitrogen removal capacity in glucose-rich environments, was immobilized within calcium alginate hydrogel beads to enhance treatment stability and reusability. The immobilized system achieved over 98% NO_3_^−^-N removal when the nitrate concentration was below 10 mg/L in simulated groundwater (Figure 7). Remarkably, the denitrification performance was consistently maintained even after five consecutive treatment cycles. When applied to real groundwater from Kumamoto City, the method effectively improved water quality, demonstrating its practical potential for real-world nitrate remediation [145].

#### 3.1.3. Radioactive Wastes

The safe management of radioactive wastes poses a significant global challenge. Hydrogels are emerging as a promising solution for this critical area. Specifically, hydrogels modified with magnetic nanoparticles or incorporating certain hexacyanoferrate complexes have demonstrated considerable potential. These specialized hydrogels can selectively remove problematic radioactive nuclides, such as Co^2+^, Cs^+^, and Sr^2+^, from contaminated wastewater primarily through highly efficient ion exchange mechanisms. Their tailored selectivity is crucial for addressing the complex nature of radioactive contamination.

Hydrogels modified with magnetic nanoparticles or hexacyanoferrate complexes have been developed for selective removal of radioactive ions such as Co^2+^, Cs^+^, and Sr^2+^ from contaminated water. These hydrogels enable efficient ion exchange and can be easily separated from treated water using magnetic fields [141].

### 3.2. Organic Pollutant Remediation

The inherent porous structure and precisely tunable surface chemistry of hydrogels make them outstanding candidates for effectively remediating a diverse range of organic contaminants found in wastewater.

### 3.3. Dyes

Industrial dyes represent a significant category of water pollutants. Their complex, often stable structures and inherent non-biodegradability pose persistent environmental challenges. Hydrogels, especially those engineered to incorporate graphene oxide or functionalized with specific cationic or anionic groups, are highly effective at adsorbing these dyes. This adsorption occurs through multiple synergistic mechanisms, including π-π stacking interactions, electrostatic interactions, and hydrogen bonding. Chitosan-based hydrogels, for instance, are particularly effective for capturing anionic dyes due to the abundance of positively charged amine groups on their surface. The remarkable efficiency of these materials is underscored by reported adsorption capacities for dyes, which can exceed 2000 mg/g [146], clearly highlighting their superior performance compared to many conventional treatment methods [14,31].

Chitosan/graphene oxide (CS/GA/RGO) composite hydrogels, further loaded with palladium nanoparticles, have demonstrated highly efficient catalytic reduction in organic dyes such as p-nitrophenol and o-nitroaniline. The synergy between chitosan’s amine groups and graphene oxide’s π-π stacking enables strong adsorption and catalytic degradation, with the composite hydrogel showing rapid and complete dye removal in model wastewater [147].

**Figure 8:**
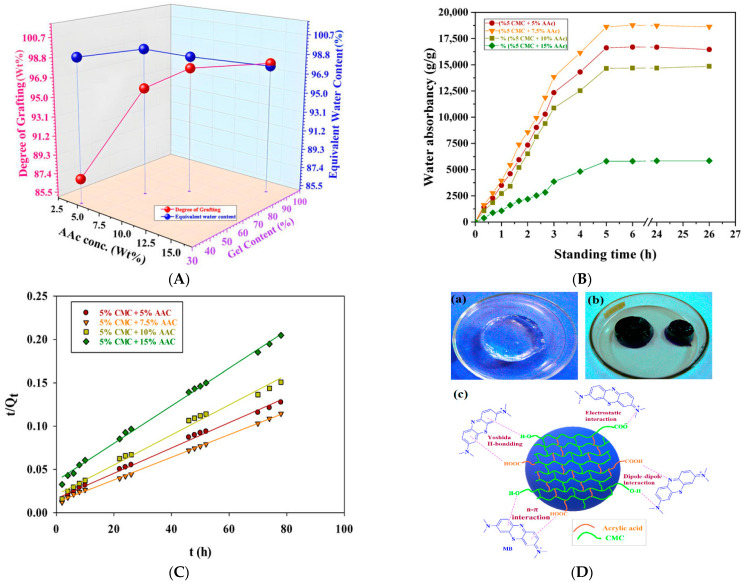
(**A**) Effect of acrylic acid concentration on the degree of grafting (Wt%), gel content (%), and EWC (%) of CMC/AAc blend hydrogels at 5 kGy radiation dose; (**B**) Effect of standing time and AAc concentration on water adsorption of CMC/AAc hydrogels at 5 kGy radiation dose; (**C**) Pseudo-second-order kinetic model for adsorption of MB on CMC/AAc hydrogel; (**D**) CMC/AAc hydrogel (**a**) before and (**b**) after MB dye adsorption and (**c**) possible adsorption mechanisms [148].

Another study focuses on the development of sustainable and efficient hydrogels for dye removal, aiming to enhance wastewater treatment technologies. CMC-based hydrogels grafted with acrylic acid (AAc) were synthesized via gamma radiation-induced polymerization. Different AAc:CMC ratios (5:5, 5:7.5, 5:10, 5:15) were treated with 37% NaOH and irradiated at doses ranging from 1 to 15 kGy, with 5 kGy producing the optimal hydrogel. Swelling tests indicated that hydrogels with 5–7.5% AAc content exhibited greater swelling, with the 5:7.5 composition reaching a maximum of 18,774.60 g/g. FTIR analysis confirmed successful grafting through interactions between AAc and CMC, while DSC results showed that increased AAc content improved thermal stability by raising both glass transition and decomposition temperatures. Enhanced grafting and reduced pore size contributed to improved swelling kinetics. The 5:7.5 hydrogel also displayed the highest adsorption capacity for methylene blue (681 mg/g at 80 mg/L), and a high desorption efficiency of 95% in 2M HCl (Figure 8). Kinetic modeling suggested the adsorption followed Schott’s pseudo-second-order model, consistent with non-uniform physisorption on a heterogeneous surface. Overall, the findings highlight a cost-effective and eco-friendly hydrogel platform suitable for large-scale water purification applications [148].

Hydrogels functionalized with various groups (e.g., cationic, anionic, or carbon-based fillers) can achieve adsorption capacities for dyes exceeding 2000 mg/g, far surpassing many conventional adsorbents. These high capacities are attributed to mechanisms such as π-π stacking, electrostatic interactions, and hydrogen bonding [149].

Natural gum-based hydrogels and their composites have also been used to remove a wide range of dyes from water, leveraging their biocompatibility and tunable surface chemistry for efficient adsorption [149,150].

**Figure 9:**
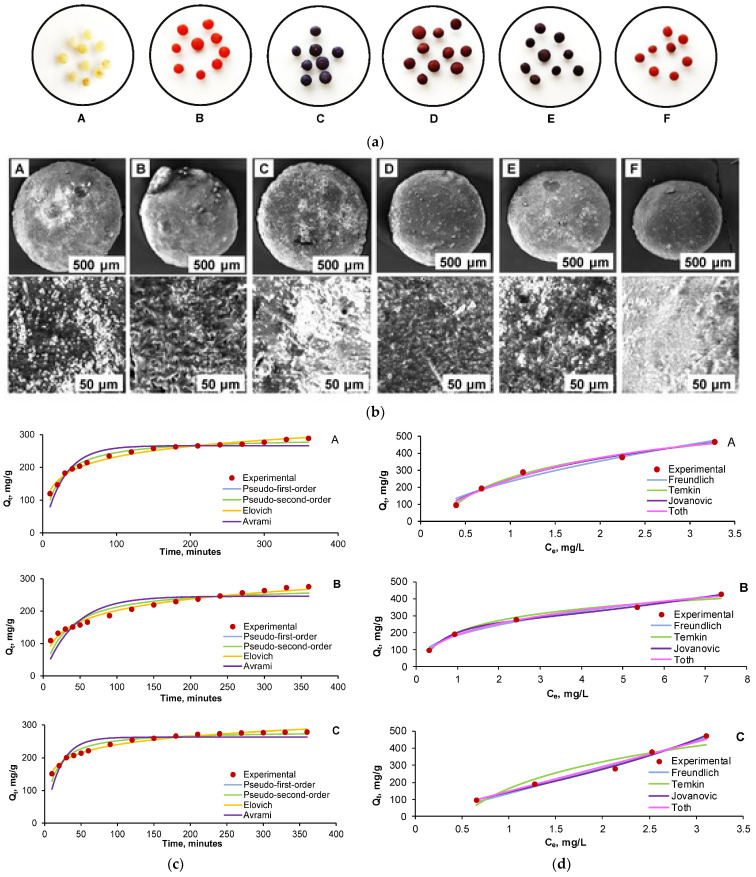
(**a**) Images of CSCH beads before dye adsorption (**A**) and after adsorption of AR (**B**), RB (**C**), 25% AR + 75% RB (**D**), 50% AR + 50% RB (**E**), and 75% AR + 25% RB (**F**); (**b**) SEM photographs of CSCH beads before dye adsorption (**A**) and after adsorption of AR (**B**), RB (**C**), 25% AR + 75% RB (**D**), 50% AR + 50% RB (**E**), and 75% AR + 25% RB (**F**). (**c**) Kinetics of AR (**A**), RB (**B**), and 50% AR + 50% RB (**C**) adsorption on CSCH adsorbent; (**d**) Equilibrium isotherms of AR (**A**), RB (**B**), and 50% AR + 50% RB (**C**) adsorption on CSCH adsorbent [151].

A recent study focused on developing a composite hydrogel by incorporating cherry stone powder into a chitosan matrix, an abundant, natural biopolymer. The resulting material (CSCH) was characterized using SEM, FTIR, and point of zero charge (pH_p_zc) analysis. The hydrogel’s adsorption performance was tested on two azo dyes, Acid Red 66 (AR) and Reactive Black 5 (RB), both individually and in a binary mixture. Process optimization was conducted using Response Surface Methodology with Central Composite Design, targeting minimal residual dye concentrations. Optimal dye removal occurred at pH 2, 100 g/L adsorbent dosage, and 30 °C, achieving over 90% removal in single dye systems and more than 70% in binary systems (Figure 8). As observed in the kinetic study, all five tested pollutant concentrations (10, 20, 30, 40, and 50 mg/L) exhibited very similar trends across both single-component systems (Acid Red 66 and Reactive Black 5) and the three binary mixtures (25% AR + 75% RB, 50% AR + 50% RB, and 75% AR + 25% RB). Therefore, for clarity and to avoid redundancy, Figure 9 presents only the equilibrium isotherms corresponding to a total initial pollutant concentration of 30 mg/L, illustrating the adsorption behavior for AR (Figure 9A), RB (Figure 9B), and their 1:1 binary mixture Overall, the composite hydrogel demonstrated strong potential as an effective, low-cost adsorbent for azo dye removal from wastewater [151].

### 3.4. Pesticides

Pesticides, such as Atrazine, are another class of persistent organic contaminants, often found in agricultural runoff. Addressing this, chitin-based hydrogels have been specifically developed and shown to be highly effective for the removal of such pesticides from agricultural wastewater, demonstrating significant adsorption capacities. This offers a promising route for mitigating the environmental impact of agricultural practices.

Photocatalytic hydrogels with high-transmission polymer networks have been developed for the degradation of glyphosate, a widely used herbicide. These hydrogels enable efficient light-driven breakdown of glyphosate at both laboratory and half-liter scales, demonstrating their potential for large-scale pesticide remediation [152].

Enzyme-assembled cellulose-based hydrogels doped with β-cyclodextrin and montmorillonite nanosheets, and further functionalized with laccase, have shown exceptional removal and degradation of polycyclic aromatic hydrocarbons (PAHs) in real wastewater, maintaining high performance even in the presence of other contaminants [153].

### 3.5. Pharmaceuticals

Pharmaceutical pollutants, such as ciprofloxacin, diclofenac, bisphenol A, and paracetamol, have emerged as persistent contaminants in aquatic environments. These compounds resist conventional wastewater treatments, accumulate in ecosystems, and potentially impact both environmental and human health. In response, scientists have developed hydrogel-based adsorbents tailored for high-efficiency removal of pharmaceuticals from water. Hydrogels are especially promising due to their three-dimensional polymeric networks, high porosity, and tunable surface chemistry, allowing them to selectively bind various pharmaceutical molecules through electrostatic interactions, hydrogen bonding, hydrophobic interactions, and chelation [154].

Table 5 presents a selection of case examples illustrating the effectiveness of various hydrogel systems in the removal of different pharmaceuticals from water.

Tetracicline: Tetracycline is a persistent environmental pollutant with low degradability, allowing it to remain in ecosystems, such as rivers, soil, and groundwater, for extended periods and circulate through the food chain, contributing to antibiotic resistance and ecological harm [166,167]. Its widespread use in veterinary medicine poses particular risk, as it is poorly absorbed in animals and accumulates in agricultural fields via excretion, with a half-life of up to 105 days [168]. Due to these risks, tetracycline is recognized as an emerging contaminant requiring effective removal strategies. For example, in a study by Kurczewska et al., composite hydrogel beads were developed using chitosan (CS) and halloysite-supported molecularly imprinted polymers (Hal@MIPa/b), synthesized under different solvent dilution conditions. The inclusion of halloysite enhanced thermal stability, with the thinner polymer layer variant (CS_Hal@MIPb) showing greater heat resistance. At optimal pH 5.0, the beads achieved high adsorption capacities (175.24–178.05 mg/g), slightly below those of free polymers but with faster equilibrium (12 h). Adsorption followed Freundlich isotherm and pseudo-second-order kinetics, and was spontaneous and exothermic. The beads demonstrated strong selectivity, maintained performance in real water samples, and improved adsorption in soil, while halloysite reduced tetracycline desorption and groundwater contamination. The primary adsorption mechanism was pore filling, supported by hydrogen bonding, π–π stacking, and electrostatic interactions [155]. In this study, a novel hydrous manganese dioxide (HMO)-modified poly(sodium acrylate) (PSA) hydrogel was developed for the simultaneous removal of tetracycline (TC) and lead (Pb(II)) from water. The composite was thoroughly characterized (SEM-EDS, FTIR, XRD, BET, XPS) to confirm successful HMO incorporation and understand its sorption mechanisms. Adsorption experiments showed rapid equilibrium within 12 h, with removal rates of 91.9% for TC and 99.5% for Pb(II). The adsorption followed pseudo-second-order kinetics; TC adsorption fit the Langmuir isotherm model, while Pb(II) followed the Freundlich model. The hydrogel showed high adsorption capacities, 475.8 mg/g for TC and 288.7 mg/g for Pb(II), with optimal removal at pH 4.0. Thermodynamic analysis revealed the adsorption was spontaneous and endothermic. These findings highlight PSA-HMO as a highly effective adsorbent for removing both antibiotics and heavy metals from contaminated water [156]. A recent study by researchers at Ewha Womans University evaluated the performance of a Fe-metal–organic framework (Fe-MOF) hydrogel composite embedded within a biopolymer-clay matrix—designated as CAMIL-MMT and CAMIL-SEP variants, for the removal of tetracycline (TC) and oxytetracycline (OTC) from aqueous environments [157]. The composite hydrogels achieved maximum adsorption capacities of 24.59 mg/g for TC and 26.14 mg/g for OTC. Kinetic modeling revealed that the adsorption followed pseudo-second-order behavior, while equilibrium data best fit the Freundlich isotherm model, indicating adsorption onto a heterogeneous surface [157].

The system showed slight sensitivity to pH, with reduced adsorption at higher pH levels; however, the presence of competing anions had minimal influence on removal efficiency. Thermodynamic analysis confirmed that the adsorption process was spontaneous and favorable. Beyond adsorption, the Fe-MOF component enabled photocatalytic degradation of residual antibiotics under visible light, enhancing the overall removal efficiency. The hydrogel also demonstrated good reusability and structural stability over multiple cycles. Although the adsorption capacities were modest compared to other high-performance hydrogels, the dual functionality of this system, adsorption and photocatalysis, combined with its stability in the presence of environmental interferences (e.g., co-existing ions), makes it a promising material for real-world applications in wastewater treatment [157]. Li et al. developed and evaluated a series of sodium alginate (SA)/polyvinyl alcohol (PVA) double-network (DN) hydrogel beads with morphology tuned by various surfactants, aiming to enhance tetracycline hydrochloride (TC) removal from water. Among the tested variants, the bead incorporating sodium dodecyl sulfate (SDS), designated SDS-B, exhibited the highest adsorption capacity of approximately 121.6 mg/g. The hydrogel beads retained high adsorption performance in the presence of competing cations and humic acid, indicating strong resistance to environmental interferences. Adsorption kinetics followed a pseudo-second-order model, suggesting chemisorption as a dominant mechanism. Isotherm analysis revealed contributions from both chemical and physical interactions. Mechanistic studies identified hydrogen bonding, electrostatic interactions, and π–π electron donor–acceptor interactions as key drivers of TC uptake. The beads demonstrated good reusability, maintaining 87.5% of their initial adsorption efficiency after three adsorption–desorption cycles. The double-network structure, combined with surfactant-induced morphological tuning, was found to enhance surface area, porosity, and active site accessibility while preserving structural integrity. This design approach yielded a hydrogel with a favorable balance between adsorption efficiency, environmental robustness, and operational sustainability, making it a viable candidate for practical antibiotic removal from contaminated water [158].

**Figure 10:**
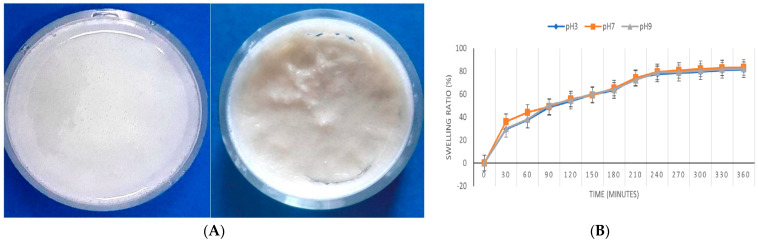
(**A**) Optical images of the CPX hydrogel: wet (**left**) and dry (**right**); (**B**) The CPX hydrogel swelling degree over 6 h at pH 3, pH 7, and pH 9 [159].

Diclofenac, a widely used anti-inflammatory drug, has a global consumption of around 940 tons annually. Approximately 65% of each oral dose is excreted through urine and feces, entering wastewater systems where conventional treatment fails to fully remove it. diclofenac also reaches water bodies through improper disposal and industrial or municipal effluents [169]. Due to its toxicity, the European Commission set strict limits for DCF concentrations: 0.01–0.1 μg/L in inland waters and 7.5–75 μg/L in coastal waters [170]. Its persistence and environmental risks have made DCF removal a growing priority aligned with several UN Sustainable Development Goals [170]. Chelu et al. developed a green hydrogel adsorbent (“CPX”) composed (Figure 10A) of chitosan (1%), PEG4000 (40%), and xanthan gum (4%) for the removal of diclofenac sodium from water. The CPX hydrogel was synthesized via a simple aqueous method and exhibited a maximum adsorption capacity of ~172.41 mg/g under optimized conditions (200 mg adsorbent, ~350 min contact). Its swelling was pH-independent (Figure 10B), and adsorption kinetics followed a pseudo-first-order model. Equilibrium data fitted both Langmuir and Freundlich isotherms, with Langmuir suggesting monolayer adsorption on uniform sites. Mechanistic studies indicate adsorption via electrostatic interactions, hydrogen bonding, and van der Waals forces. Although the hydrogel shows strong performance and eco-friendly synthesis, the relatively long equilibration time and limited information on regeneration cycles are potential drawbacks [159].

Bisphenol compounds such as bisphenol A (BPA), bisphenol S (BPS), and bisphenol F (BPF) are widely used in the plastics industry and are frequently detected in water sources. Even at low concentrations, they exhibit endocrine-disrupting effects and have been linked to cardiovascular, reproductive, and neurobehavioral disorders [171]. This highlights the urgent need for effective methods to remove these organic micropollutants from water. Lv et al. developed a porous nanofibrous membrane composed of cellulose acetate modified with β-cyclodextrin-containing polymers (CA–P–CDP) for the rapid and efficient removal of trace bisphenol pollutants, including BPA, BPS, and BPF, from water. The membrane exhibited a highly porous structure, good thermal stability, and enhanced surface functionality due to β-cyclodextrin grafting [160]. Under optimal conditions (pH 7.0, 25 °C, 0.1 g/L dosage), the membrane achieved maximum adsorption capacities of 50.37 mg/g (BPA), 48.52 mg/g (BPS), and 47.25 mg/g (BPF), with adsorption equilibrium reached within 15 min. Adsorption kinetics followed a pseudo-second-order model, indicating chemisorption as the rate-controlling step. Equilibrium data were best described by the Liu isotherm model, suggesting a combination of monolayer and heterogeneous adsorption. Mechanistically, the adsorption process was driven by hydrophobic interactions, hydrogen bonding, and π–π stacking between the bisphenol molecules and functional sites on the modified membrane. The membrane demonstrated strong performance in dynamic flow systems, treating up to 0.58 L of BPA-contaminated water, over 14 times the volume treated by unmodified cellulose membranes. High removal efficiencies were also maintained in real water matrices such as lake and river water, highlighting the practical applicability of the CA–P–CDP membrane in removing endocrine-disrupting bisphenol pollutants from complex aqueous environments [160].

Ciprofloxacin, a broad-spectrum fluoroquinolone antibiotic, is frequently detected in aquatic environments due to its widespread use in human and veterinary medicine, improper disposal, and excretion. It is considered a contaminant of emerging concern due to its persistence, incomplete degradation during wastewater treatment, and potential to contribute to antimicrobial resistance. Environmental studies have confirmed the occurrence of ciprofloxacin in wastewater, surface water, and even drinking water source [172]. Ecotoxicological effects include disruption of microbial communities and promotion of antibiotic resistance genes. Due to its pseudo-persistent behavior and ecological risk, ciprofloxacin requires urgent remediation strategies. Recent approaches include advanced oxidation processes, biochar adsorption, and hydrogel-based composites, which have shown promise in efficiently removing CIP from contaminated water. Konwar et al. developed a magnetic alginate–Fe_3_O_4_ hydrogel fiber for the removal of ciprofloxacin hydrochloride from aqueous solutions [161]. The fibers were fabricated using a wet-spinning technique in which alginate hydrogel was embedded with Fe_3_O_4_ magnetic nanoparticles. Structural and compositional analyses confirmed successful integration of the nanoparticles via FTIR, XRD, and SEM, while thermal and mechanical stability were assessed through TGA and tensile strength measurements. Magnetic properties were characterized using vibrating sample magnetometry. The incorporation of Fe_3_O_4_ significantly enhanced ciprofloxacin adsorption compared to non-magnetic alginate fibers, which exhibited minimal uptake. The magnetic nature of the composite enabled easy separation and recovery from solution using an external magnetic field. Moreover, the presence of Fe_3_O_4_ improved both the thermal and mechanical stability of the hydrogel fibers, attributed to strong interactions between the polymer matrix and the nanoparticles. Although mechanistic analysis was limited, the adsorption was inferred to be governed by electrostatic interactions and possibly hydrogen bonding between the drug molecules and active sites on the hydrogel and nanoparticle surfaces. The combination of enhanced stability, adsorption capacity, and reusability highlights the potential of this composite for effective antibiotic removal from water systems [161]. Rasoulzadeh et al. synthesized a magnetite-imprinted chitosan nanocomposite (Fe–CS NC) for the selective adsorption of ciprofloxacin from aqueous solutions [162]. The material was designed to enhance molecular recognition and surface affinity for the target antibiotic by incorporating Fe_3_O_4_ nanoparticles into a chitosan matrix. The composite was characterized using SEM, TEM, XRD, FTIR, and BET surface area analysis, with zeta potential measurements used to evaluate surface charge and interaction potential. Response surface methodology (RSM) was employed to optimize operational parameters including pH, adsorbent dose, and contact time. The nanocomposite achieved a maximum adsorption capacity (q_m_) of approximately 142 mg/g under optimal conditions, with a removal efficiency of around 68%. Kinetic studies revealed that the adsorption process followed a pseudo-second-order model, indicating chemisorption as the dominant mechanism. Isotherm analysis showed the best fit with the Freundlich model, suggesting heterogeneous surface adsorption. The adsorption was highly pH-dependent: efficiency was reduced at pH values below the chitosan isoelectric point due to protonation of amine groups, while optimal performance occurred near pH 6, where electrostatic attraction and hydrophobic interactions were maximized. Thermodynamic analysis confirmed that the adsorption process was spontaneous and endothermic. The material also demonstrated good reusability, maintaining performance over multiple adsorption–desorption cycles. These findings support the potential of Fe–CS NC as an efficient and regenerable adsorbent for ciprofloxacin removal from water systems [162].

Ibuprofen, a widely used non-steroidal anti-inflammatory drug, has emerged as a significant micropollutant in aquatic environments due to its high global consumption and partial removal in conventional wastewater treatment plants. It is frequently detected in surface waters, sewage effluents, and even drinking water, primarily entering water systems through human excretion and improper disposal of pharmaceuticals [173]. Studies show that the primary transformation products of ibuprofen, such as hydroxy- and carboxy-ibuprofen, can persist in the environment and may even surpass the concentration of the parent compound in treated wastewater and rivers [173]. This hydrogel system was designed to remove ibuprofen via adsorption using a composite of natural polymers (alginate and carboxymethyl cellulose) reinforced with activated carbon. The initial adsorption capacity was 48.1 mg/g, indicating good affinity toward ibuprofen. However, upon reswelling (i.e., rehydration after drying), the adsorption performance significantly decreased to 18% of the original capacity (q_e_ = 8.6 mg/g). This sharp decline is likely due to structural changes or reduced accessibility of active sites after drying and reswelling. The poor recovery in adsorption performance raises concerns about the reusability and regeneration efficiency of this hydrogel system, limiting its practical application in water treatment unless modifications are introduced to enhance structural stability [164]. In this system, GO and PANI nanoparticles were embedded into a chitosan hydrogel matrix to adsorb ibuprofen. However, the study found that as the GO content increased, the adsorption efficiency for ibuprofen decreased. This may be attributed to excessive GO causing aggregation, reducing surface area, or altering the surface chemistry in a way that diminishes ibuprofen binding. Despite the promising multifunctionality of GO and PANI for pollutant removal, the results suggest that optimization of nanoparticle loading is critical to balance structural enhancement with adsorption performance. Overloading with GO can be detrimental, emphasizing the importance of formulation control [165].

### 3.6. Microbial Disinfection

Some hydrogels naturally possess antimicrobial properties, presenting a promising pathway for microbial disinfection in water treatment.

Antimicrobial hydrogels are a crucial innovation for ensuring safe drinking water. These hydrogels can be loaded with different nanoparticles, which are well-known for their potent antimicrobial effects. Alternatively, certain hydrogels, particularly those derived from chitosan, have inherent antimicrobial properties due to their natural structure. Both approaches lead to the effective inactivation of harmful bacteria and other microorganisms present in water, contributing significantly to public health and safety.

#### 3.6.1. Chitosan-Based Hydrogels (Inherent Antimicrobial Activity)

Chitosan-based hydrogels are gaining significant attention for water disinfection due to their inherent antimicrobial properties, biocompatibility, and structural versatility. Their polycationic nature enables them to disrupt microbial membranes, making them effective against a wide range of pathogens. Recent advancements have further improved their antimicrobial efficacy through compositional modifications and the incorporation of functional additives. One such innovation involves the integration of plasma-activated water (PAW) into chitosan hydrogels. The reactive oxygen and nitrogen species (RONS) generated in PAW enhance the antimicrobial potential of the hydrogel system. As demonstrated by Cuellar-Gaona et al. chitosan hydrogels containing PAW exhibited significantly enhanced antibacterial and antifungal activity, along with excellent hemocompatibility, making them promising candidates for safe and effective water disinfection applications [174]. Comprehensive reviews also highlight chitosan’s effectiveness in inactivating a broad spectrum of waterborne pathogens [175].

In practical water treatment scenarios, chitosan nanoparticles (CNs) have also proven highly effective. Deņisova et al. reported that medium molecular weight chitosan NPs at 0.25% concentration achieved over 99.99% reduction in *E. coli* from tap water. This highlights the potential of CNs as fast-acting and biodegradable disinfectants in real-world water purification systems [176].

A broader perspective on chitosan’s utility in water purification was provided by Chelu et al., who reviewed its use in hydrogel form across various water treatment applications. They emphasized chitosan’s biocompatibility, environmental safety, and natural antimicrobial capabilities, which make it an ideal matrix for developing eco-friendly disinfection systems [177].

Finally, quaternary ammonium-modified chitosan hydrogels have emerged as potent antimicrobial materials. Andreica et al. reported that these hydrogels demonstrated strong antibacterial activity, excellent hemocompatibility, and biodegradability. Their skin adhesivity and safety profile make them attractive not only for water disinfection but also for biomedical and wound-healing applications [178].

#### 3.6.2. NPs-Loaded Hydrogels

Nanoparticle-loaded hydrogels have emerged as powerful materials for water disinfection due to their enhanced antimicrobial efficacy, biocompatibility, and tunable release profiles. These systems integrate the broad-spectrum antimicrobial properties of metallic or metal oxide nanoparticles (NPs) with the physical and chemical advantages of hydrogels, enabling effective microbial decontamination across diverse water environments.

Ag NPs are among the most widely studied for hydrogel-based antimicrobial applications. Their integration into hydrogels facilitates sustained release of Ag^+^ ions, which disrupt bacterial membranes and inhibit replication. For instance, AgNP-infused hydrogels have demonstrated synergistic properties for wound healing and disinfection, making them promising candidates for water sanitation as well. These systems exhibit broad-spectrum efficacy and can be tailored for targeted applications. [179,180]. Advanced hydrogels combining silver and gold NPs also show promise in combating multidrug-resistant bacteria. These composites benefit from the high retention capacity and moisture of hydrogels while allowing precise NP delivery to microbial targets [181].

Titanium dioxide (TiO_2_)-based hydrogels are also notable for their photocatalytic properties. For instance, TiO_2_ NPs embedded in carboxymethyl cellulose (TiO_2_NPs@CMC) hydrogels achieved complete inactivation of Salmonella typhi and E. coli O157 within 180 min under light exposure, offering sustainable wastewater disinfection capabilities with minimal toxicity [182]. Hydrogels containing TiO_2_ NPs in combination with graphene platelets also show promise, enabling fast photocatalytic degradation of contaminants while maintaining mechanical integrity and porosity. These materials allow pollutant molecules to freely diffuse and degrade within the hydrogel matrix [183].

Magnetic hydrogel microbots (MHMs), made by coating hydrogel droplets with multifunctional magnetic nanoparticles, offer dynamic, rapid decontamination in both static and flow-through systems. These MHMs remove 95% of organic pollutants within 3 min and are capable of self-regeneration via environmentally friendly H_2_O_2_ precursors [184].

Another unique approach includes the development of natural seed-based hydrogel bits loaded with Fe_3_O_4_ or N-doped TiO_2_ for multifunctional water purification, including microbial and heavy metal ion removal. These gel-bits can be magnetically recovered or filtered easily, avoiding issues related to free nanoparticle dispersion [185].

Beyond silver, zinc oxide (ZnO) NPs incorporated into gum acacia/polyacrylate hydrogels have been synthesized in situ and proven effective against *E. coli*, showing enhanced water absorption and antimicrobial action [185].

Graphene-based hydrogels also play a vital role in disinfection technologies. Functionalized graphene nanomaterials embedded in hydrogel matrices exhibit strong antibacterial properties due to mechanical membrane disruption and oxidative stress, as detailed in recent reviews [186].

Other systems include:

- AgNPs-loaded acacia gum/chitosan nanogels used for coating pipes to inhibit microbial adhesion and biofilm formation. These coatings significantly reduced contamination in water distribution systems and proved to be non-toxic [169,187].
- Hydrogels with lysozyme immobilized demonstrated long-term antibacterial activity against *E. coli* and *B. subtilis*, offering a safer alternative to metallic NP systems [188].
- In situ-formed AgNPs in carbohydrate-based supramolecular hydrogels created efficient antimicrobial matrices without external reducing agents, utilizing glucosaminyl-barbiturate chemistry [189].

### 3.7. Desalination and Oil–Water Separation

Hydrogels are also being explored for more specialized water treatment applications, extending their utility beyond traditional pollutant removal.

One promising area is desalination. Salt-responsive hydrogels are showing potential for use in forward osmosis and other low-energy desalination techniques. While still an emerging field, this application could offer more sustainable and energy-efficient ways to produce fresh water.

Another significant area is oil–water separation. Superabsorbent hydrogels can be engineered with specific surface properties, either oleophilic/hydrophobic (oil-attracting/water-repelling) or hydrophilic/oleophobic (water-attracting/oil-repelling). This tailored design allows them to efficiently separate oil from oily wastewater, making them highly valuable for managing industrial effluents or responding to large-scale oil spills.

Hydrogels are being developed for advanced water treatment applications such as desalination and oil–water separation, offering innovative solutions beyond conventional pollutant removal.

Table 6 highlights some examples of hydrogel systems and their impressive performance in the specialized applications of oil–water separation and desalination.

#### 3.7.1. Desalination

Salt-responsive hydrogels are emerging as sustainable and efficient materials for water desalination, particularly through solar-driven evaporation and interfacial water purification. These systems utilize hydrophilic polymer networks embedded with photothermal materials to enhance water absorption, solar energy conversion, and salt rejection

While this field is still developing, some hydrogel systems have demonstrated the ability to selectively absorb water and reject salts, making them suitable for sustainable freshwater production. For example, composite hydrogels incorporating photothermal materials like carbon black have been used for solar-driven water purification, where the hydrogel platform enables efficient solar distillation and clean water recovery from saline sources [195]. These approaches highlight the potential of hydrogels to contribute to energy-efficient desalination technologies.

One prominent example is a PVA-based hydrogel integrated with reduced graphene oxide (rGO), which achieved a high evaporation rate of 2.5 kg m^−2^ h^−1^ under 1 sun irradiation. The rGO served as a solar absorber, while the hydrophilic PVA framework facilitated water transport and reduced evaporation enthalpy, enabling long-term desalination and antifouling performance [196].

Another advanced system involves dual-crosslinked cellulose-based hydrogels doped with iron-based metal–organic frameworks. These structures provided strong light absorption and achieved an evaporation efficiency of 89.32%, effectively purifying seawater and wastewater under real sunlight conditions [197].

Gradient-charged hydrogels also show promise by creating osmotic pressure differentials that enhance water transport. These systems demonstrated efficient evaporation even in high-salinity environments like 20% NaCl solutions, supporting the removal of salts and other impurities from brine [198].

A noteworthy all-natural solution features a hydrogel composed of chitosan, cellulose nanofibers, and carbonized spent bleaching earth (C@SBE). This system exhibited strong photothermal performance and achieved an evaporation rate of 2.17 kg m^−2^ h^−1^, alongside heavy metal removal capabilities, including Cu^2+^ uptake up to 145.3 mg/g [199].

For brine desalination in the oil and gas industry, nanogel materials composed of poly(vinyl alcohol) and polypyrrole enhanced solar absorption, resulting in desalination rates up to 4.5 times higher than untreated samples, and over 99% ion removal [200].

#### 3.7.2. Oil–Water Separation

Hydrogels have shown remarkable versatility in oil–water separation, especially when engineered with tailored surface properties:

A Lantern [33]arene-based supramolecular hydrogel was developed with a low critical gelation concentration and used to coat stainless-steel mesh. This hydrogel-coated mesh achieved oil–water separation efficiency greater than 99% and high flux (>60,000 L m^−2^ h^−1^). The system is also pH-responsive and can be reused, demonstrating both high performance and adaptability for industrial oil–water separation [190].

A supramolecular nanofibrous hydrogel membrane with a core–shell structure was fabricated for oil-in-water emulsion separation, even under strong acidic conditions (pH = 1). The membrane maintained high separation permeance (over 17,000 L m^−2^ h^−1^ bar^−1^) and efficiency after hours of continuous operation, making it suitable for large-scale filtration in harsh environments [191].

A biodegradable starch/PVA hydrogel was engineered to selectively absorb water from oil–water emulsions, exhibiting underwater oleophobicity (oil contact angle ~153.6°) and high biodegradability (~90% in 28 days). This hydrogel is promising for both environmental remediation and sustainable product development [192].

A double network hydrogel coating with tannic acid and graphitic carbon nitride (g-C_3_N_4_) was applied to membranes, achieving separation fluxes over 40,000 L m^−2^ h^−1^ and efficiency above 99%. The membrane also featured photocatalytic self-cleaning, maintaining performance over 40 cycles and enabling long-term use in oily wastewater purification [193].

Physically crosslinked cellulose hydrogels were used as coatings on stainless mesh, providing underwater superoleophobicity and separation efficiency above 98.9%. The system maintained high flux and reusability, even in saline conditions, demonstrating the sustainability and robustness of biomass-derived hydrogels [194].

## 4. Challenges and Future Perspectives

Despite the significant advancements in sustainable hydrogel development, several challenges must be addressed for their widespread adoption in water treatment [13]

One major limitation is mechanical strength. Many natural polymer-based hydrogels, while biodegradable, often suffer from low mechanical strength. This can restrict their use in high-flow or pressure systems, potentially leading to structural instability and reduced reusability [201].

Another concern is saturation kinetics. The adsorption capacity of hydrogels will eventually become saturated, and the rate at which this occurs, along with their maximum capacity, directly influences their practical utility in continuous treatment processes [202].

Furthermore, interference from multi-component systems is a critical issue. Real wastewater is a complex mixture of various pollutants, yet most research focuses on single-solute systems. The performance of hydrogels in these complex multi-component adsorption systems requires much more thorough investigation. Finally, the long-term stability and consistent performance of hydrogels under diverse real-world operating conditions, including varying pH, temperature, ionic strength, and the presence of other contaminants, require extensive further validation to ensure their reliability [203].

Despite their promising potential, the practical application of sustainable natural hydrogels is constrained by several key limitations that require further research and development. A significant challenge lies in reusability and regeneration. While the scientific literature demonstrates successful pollutant removal over a few cycles, achieving multiple, effective regeneration cycles in complex wastewater matrices remains difficult. Each cycle can lead to a gradual loss of adsorption capacity due to irreversible binding or structural degradation. Furthermore, the selective adsorption of target pollutants is not always guaranteed. In real wastewater, which is a multi-component system, hydrogels may face competitive adsorption from co-existing ions, which can significantly reduce their efficiency and selectivity for the target contaminant. Finally, there is a delicate balance between biodegradability and long-term stability. While the natural origin of these hydrogels makes them inherently biodegradable, their lifespan must be sufficient to perform their function without premature breakdown. Conversely, if the degradation is too slow, the hydrogel could persist in the environment as a form of secondary pollution, especially if it retains adsorbed contaminants. Addressing these fundamental limitations is crucial for moving these innovative materials from the laboratory bench to industrial-scale viability.

Circular economy approaches are also paramount. This includes waste-derived precursors, where further research into utilizing diverse waste streams (such as agricultural waste, industrial byproducts, and discarded plastics) as precursors for hydrogel synthesis can dramatically enhance their sustainability and economic viability [15]. Equally important are recycling strategies. Developing robust and energy-efficient methods for spent hydrogels, especially those with encapsulated inorganic components, is critical to avoid creating new waste problems. This involves exploring chemical, thermal, or biological methods to recover valuable components or regenerate the adsorbent for reuse [92].

Scaling up the production of natural hydrogels for industrial water treatment presents significant economic challenges that extend far beyond laboratory-scale costs. While these materials are derived from low-cost, abundant sources, the journey to commercial viability involves a detailed examination of several key factors [61].

The primary cost drivers are the raw materials and the synthesis process. Although natural polymers like chitosan from crustacean shells or cellulose from wood pulp are inexpensive, the cost of processing them into high-purity, standardized precursors can be considerable. The synthesis process itself, while often termed “green,” can be complex. Techniques like solvent-free or microwave-assisted crosslinking may reduce environmental impact, but they often require specialized equipment that is more expensive than conventional reactors, leading to high capital expenditure. Furthermore, ensuring consistent material properties, such as porosity, swelling capacity, and mechanical stability, across large-scale batches is a technical challenge that can increase production costs due to the need for stringent quality control and more sophisticated process monitoring.

Another critical economic factor is the long-term viability of the hydrogels, which is directly tied to their regeneration and recycling. The ability to reuse the hydrogels multiple times is essential for making them economically competitive with disposable sorbents. However, the costs associated with the regeneration process, including the energy required for desorption and drying, must be carefully considered. While some regeneration methods are simple, others can involve chemical agents that add to operating costs and introduce secondary waste streams. For a true economic and environmental comparison, lifecycle assessment studies are crucial. Navigating regulatory requirements for environmental safety and efficacy will also be essential for successful market entry [14]. These studies quantify the total costs and energy consumption from raw material extraction to final disposal, providing a holistic view of a hydrogel’s economic and environmental footprint. This comprehensive analysis helps determine if the initial high costs of green synthesis are offset by long-term savings from reusability and reduced waste, thereby proving the overall economic viability of these sustainable materials for industrial applications.

Despite these limitations, exciting innovation opportunities are emerging that can propel sustainable hydrogels forward. Artificial intelligence-driven hydrogel design is a transformative prospect; artificial intelligence and machine learning can revolutionize hydrogel development by predicting optimal material compositions, synthesis parameters, and functionalization strategies for specific pollutant removal. This can significantly accelerate the discovery of novel, high-performing, and sustainable hydrogels [204].

Furthermore, the integration with advanced technologies holds great promise. Combining hydrogels with other cutting-edge treatment technologies, such as membrane filtration (e.g., hydrogel-enhanced membranes for improved antifouling) or electrochemical processes, could lead to synergistic effects and significantly enhance overall water purification performance [31]. Lastly, the development of smart and responsive hydrogels presents a frontier for innovation. Designing hydrogels that can respond to external stimuli like light or magnetic fields for on-demand adsorption/desorption or possess self-healing properties would open entirely new avenues for highly efficient and automated water treatment systems [205].

## 5. Conclusions

Polymeric hydrogels, particularly those derived from sustainable and natural sources, represent a transformative solution for advanced water treatment, offering distinct advantages over conventional methods. This review has systematically demonstrated how these materials are paving the way for a more sustainable and effective approach to environmental remediation.

The core of this sustainability lies in the use of abundant and biodegradable natural polymers like chitosan, cellulose, and alginate, which provide the foundational framework for hydrogel systems. These biopolymers possess inherent functional groups that enable efficient pollutant capture, while a shift towards green synthesis routes—including solvent-free, microwave-assisted, and bio-derived crosslinking methods—further reduces their environmental footprint by minimizing the use of toxic chemicals and lowering energy consumption.

As discussed, through innovative functionalization strategies and the creation of nanocomposite hydrogels, these materials can be precisely engineered to selectively remove a wide range of pollutants, from heavy metals like Pb^2+^ and Cr^6+^ to organic dyes and radioactive wastes such as Cs^+^ and Sr^2+^. Their high adsorption capacities, easy separation via magnetic or filtration methods, and potential for regeneration confirm their high performance and reusability.

Despite existing challenges, such as mechanical limitations and scalability hurdles, the future of sustainable hydrogels is promising. Ongoing research and emerging technologies, including.

AI-driven material design, waste valorization, and pilot-scale validation, are poised to address these issues and accelerate the transition from laboratory prototypes to practical, industrial applications. Ultimately, the integration of these eco-friendly materials into a circular economy framework will lead to truly effective and responsible water purification technologies for a healthier planet.

## Figures and Tables

**Table 1:** Comparison of hydrogel raw materials for environmental applications.

Material	Source	Key Functional Groups	Target Pollutants	Sustainability Pros (✓)/Cons (✗)	References
Chitosan	Crustacean shells, fungi	–NH_2_, –OH	Heavy metals (Cu^2+^, Cr^6+^), anionic dyes, microbes	✓ Biodegradable, renewable; ✗ Limited mechanical strength, Batch variability	[84]
Cellulose	Plants, bacteria (e.g., cotton, wood)	–OH	Dyes, heavy metals (Cd^2+^, Ni^2+^), organics	✓ Abundant, renewable, biodegradable; ✗ Often requires modification for specific binding	[85]
PVA	Synthetic	–OH	Broad-spectrum (e.g., heavy metals Ni^2+^, Cu^2+^, Co^2+^, Cr^6+^), dyes, radionuclides	✓ High durability, tunable properties; ✗ Slow biodegradation, petroleum-derived	[86]
Alginate	Brown seaweed	–COO^−^	Divalent metals (Pb^2+^, Cd^2+^, Cu^2+^), dyes	✓ Mild processing, Biodegradable, Renewable; ✗ Lower mechanical strength than synthetics	[87]
PAA	Synthetic	–COOH	Cationic dyes, various metal ions (Cd^2+^, Fe^3+^)	✓ High swelling, precise functionality; ✗ Non-biodegradable (unless modified), toxic monomer (acrylamide)	[88]
Starch	Plants (e.g., corn, potato)	–OH	Dyes (e.g., methylene blue), heavy metals (Pb^2+^), organics	✓ Low-cost, abundant, biodegradable; ✗ Low mechanical strength, limited selectivity	[89]
Lignin	Pulp and paper industry byproduct	Phenolic –OH, –COOH	Heavy metals, dyes	✓ Waste-derived, renewable, biodegradable; ✗ Complex structure, requires modification	[90]
Soy protein	Soybeans	–NH_2_, –COOH, –SH	Heavy metals (Cd^2+^, Pb^2+^), specific organics	✓ Waste-derived, biodegradable; ✗ Mechanical weakness, batch variability	[91]
Guar gum	Guar beans	Numerous hydroxyl (-OH) groups	Heavy metals, dyes, and specific pollutants like boron. Its hydroxyl groups facilitate adsorption through hydrogen bonding and complexation	✓ It is a low-cost, food-grade natural product that is biodegradable and non-toxic. Its production is relatively simple and requires less energy compared to synthetic polymers. ✗ Guar gum-based hydrogels may have lower mechanical stability in certain conditions and can be susceptible to microbial degradation, which could limit their long-term use in some applications.	[33]
Pectin	Citrus peels, apple pomace, and sugar beet pulp (by-products of the food industry)	Abundant carboxyl (-COOH) groups and hydroxyl (-OH) groups.	Highly effective for removing heavy metal ions and radioactive ions (e.g., Sr^2+^ and Cs^+^) via ion exchange and chelation with its carboxyl groups.	✓ It is a cost-effective, non-toxic, and biodegradable material derived from food waste, which promotes a circular economy. ✗ Pectin-based hydrogels can have low mechanical strength and stability, which may require modification or combination with other materials for practical applications	[34]

**Table 2:** Comparison of green synthesis methods for hydrogels.

Method	Reaction Time	Energy Use	Scalability	Best for
Solvent-free	Moderate (1–4 h)	Low	High	Thermoplastic polymers (PVA, starch), bulk production [96]
Microwave-assisted	Fast (minutes)	Very low	Moderate	Grafting, nanocomposite formation, rapid prototyping [97]
Bio-derived crosslinkers	Moderate (2–6 h)	Low	High	Natural polymers (chitosan, alginate), biocompatible applications [98]
Radiation-induced	Fast (seconds–minutes)	Moderate	Low to moderate	High-precision networks, sterile products, unique grafting [99]

**Table 3:** Characterization techniques.

Category	Technique	Purpose	Advantages	Limitations	Typical Applications	References
Physicochemical	Fourier transform infrared spectroscopy (FTIR)	Identify chemical bonds and functional groups	Confirms crosslinking/functionalization	Overlapping peaks, limited quantification	Chemical structure verification	[132,133,134]
	Scanning Electron Microscopy (SEM)	Visualize surface morphology and pore distribution	High-resolution imaging	Requires dehydration, may alter hydrogel	Morphology–performance correlation	
	Thermogravimetric Analysis/Differential Scanning Calorimetry (TGA/DSC)	Assess thermal stability and transitions	Provides decomposition profiles	Requires dry samples, not in situ	Predicting durability under heat	
Analytical (drug detection/quantification)	UV-Vis	Drug quantification (small molecules)	Cheap, fast	Low sensitivity, poor selectivity	Routine release assays	
	Fluorescence	Detect drugs/proteins (often tagged)	High sensitivity, imaging capability	Requires labeling, possible artifacts	Protein/nucleic acid tracking	
	Enzyme-Linked Immunosorbent Assay (ELISA)	Protein quantification	Highly selective and sensitive	Interference issues, costly kits	Growth factor release studies	
	High-Performance Liquid Chromatography MS (HPLC)	Separate and quantify drugs	High precision and sensitivity	Time-consuming, expensive	Small molecule drugs, pharmacokinetics	
	Mass Spectrometry (MS)	Detect and quantify diverse molecules	Ultra-sensitive and specific	Costly, expertise needed	Proteins, peptides, metabolites	
	Polymerase Chain Reaction/Reverse Transcription quantitative (PCR/RT-qPCR)	Nucleic acid quantification	Sensitive, gold standard for DNA/RNA	Expensive, complex	Gene/drug delivery efficacy	
Drug diffusion	Franz Cell Assay	Diffusion measurement	Physiologically relevant	Limited to membrane studies	Transdermal delivery	
	Fluorescence Recovery After Photobleaching/Fluorescence Correlation Spectroscopy (FRAP/FCS)	Fluorescent mobility studies	Real-time, microscale	Requires labeling	Drug/protein dynamics in hydrogels	
	Microfluidics	High-throughput diffusion studies	Real-time, small sample sizes	Specialized setup	Advanced screening of hydrogel systems	
Biological evaluation	In vitro models	Biocompatibility and efficacy	Controlled conditions	Simplified environment	Initial screening of formulations	
	In vivo models	Pharmacokinetics, toxicity	Physiological relevance	Ethical, costly, complex	Preclinical validation	

**Table 4:** Case examples of hydrogel applications for pollutant removal.

Pollutant Type	Hydrogel System and Functionalization	Adsorption Capacity (mg/g)	Removal Efficiency (%)	Reference
Pb(II)	Alginate/coffee waste composite	Up to 199.2 mg/g	98.4% removal, reusable	[135]
Co(II)	Modified starch hydrogel from a copolymer of 2-acrylamido-2-methylpropane sulfonic acid (AMPS) and N,N-dimethylaminoethyl methacrylate (DMAEMA)	350 mg/g	Up to 98.76% removal	[136]
Phosphate (PO_4_^3−^)	Modified starch hydrogel (AMPS)	650 mg/g	Exceeding 90%	[136]
Cr(VI)	Grafted cellulose hydrogel from a copolymer of acrylamide (AM) and sodium polyacrylate (SPA)	Up to 139.40 mg/g	90–96% removal	[137]
Multiple heavy metals	Chitosan/cellulose/alginate-based composites	38–440+ mg/g	Up to 93% removal	[123]
Radioactive ions	Magnetic nanoparticle/hexacyanoferrate hydrogels	For ex, for Strontium (Sr^2+^) is 421.94	High selectivity, easy recovery	[138]

**Table 5:** Case examples of hydrogel applications for pharmaceutical pollutant removal.

Pollutant	Hydrogel System	Key Properties	Reference
Tetracycline	Chitosan-halloysite molecularly imprinted hydrogel	Selective, high thermal stability, max adsorption ~178 mg/g	[155]
	Hydrous manganese dioxide-poly(sodium acrylate)	High adsorption (~476 mg/g), fits Langmuir model, pH 4 optimal	[156]
	Fe-MOF/biopolymer-clay hydrogel	Adsorption ~24.6 mg/g, pseudo-second-order kinetics	[157]
	Sodium alginate/PVA hydrogel	High removal (121.6 mg/g), good reusability	[158]
Diclofenac	Chitosan-PEG-Xanthan hydrogel	Green synthesis, biodegradable, 172.41 mg/g capacity	[159]
Bisphenol A	β-cyclodextrin modified cellulose nano-fiber membrane	Maximum adsorption capacities were 50.37, 48.52 and 47.25 mg g^−^	[160]
Ciprofloxacin	Magnetic alginate–Fe_3_O_4_ hydrogel	Magnetic, recyclable, high efficiency	[161]
	Magnetite imprinted chitosan polymer nanocomposites	Maximum adsorption capacity was obtained 68% and 142 mg/g,	[162]
Paracetamol	Cross-linked chitosan supported bimetallic-oxide nanoparticles, specifically ZnO and Fe_3_O_4_	Removal efficiency of ~99% (q_m_ = 4.98 mg g^−1^), with a Zn:Fe mole ratio of 1:1.	[163]
Ibuprofen	Reswellable alginate/activated carbon/carboxymethyl cellulose hydrogel beads	The reswelled beads exhibited only 18% (q_e_ = 8.6) of the initial adsorption capacity (q_e_ = 48.1)	[164]
	GO and PANI nanoparticles rationally immobilized in chitosan matrix-based hydrogel	The adsorption of ibuprofen reduces as the quantity of GO in the thin films increases	[165]

**Table 6:** Hydrogel applications in oil–water separation and desalinization.

Application Area	Hydrogel System and Key Feature	Performance/Outcome	Citations
Oil–water separation	Lantern [33]arene supramolecular hydrogel	>99% efficiency, >60,000 L m^−2^ h^−1^	[190]
	Acid-resistant nanofibrous hydrogel membrane	>17,000 L m^−2^ h^−1^ bar^−1^, acid stable	[191]
	Starch/PVA superabsorbent hydrogel	Oleophobic, 90% biodegradability	[192]
	Gelatin-based double network hydrogel	>99% efficiency, self-cleaning	[193]
	Cellulose hydrogel coating	>98.9% efficiency, saline stable	[194]
Desalination/solar distillation	Chitosan/PVA/carbon black hydrogel platform	Solar-driven water purification	[195]

## Data Availability

No new data were created or analyzed in this study. Data sharing is not applicable to this article.
